# Supplementary material for: Higher-order topological insulator in cubic semiconductor quantum wells
Source: Sci Rep. 2021 Oct 26;11:21060. doi: 10.1038/s41598-021-00577-z (PMC8548307; doi:10.1038/s41598-021-00577-z)
Supplement: Supplementary file 1 — Supplementary Information. [file 41598_2021_577_MOESM1_ESM.pdf]

Supplementary Materials for

Higher-order topological insulator in cubic semiconductor quantum wells

Sergey S. Krishtopenko[1,2,*]

[1] *CENTERA Laboratories, Institute of High Pressure Physics, Polish Academy of Sciences, PL-01-142 Warsaw, Poland*

[2] *Laboratoire Charles Coulomb (L2C), UMR 5221 CNRS-Université de Montpellier, F- 34095 Montpellier, France*

This PDF file includes:

- Supplementary Text;
- Fig. S1. Parameters of effective 1D edge Hamiltonian as a function of the edge orientation φ for the three-layer InAs/GaInSb and double HgTe/CdHgTe QWs considered in the main text at different growth orientations. The edge independent parameters (v_0, v_z) equal to (36.6, 68.1) meV·nm and (−16.9, 200.3) meV·nm for the three-layer InAs/GaInSb and double HgTe/CdHgTe QWs, respectively;
- Table S1. Parities of the envelope functions of multi-band $\mathbf{k}\cdot\mathbf{p}$ Hamiltonian at zero electron momentum in the QW plane;
- Table S2. Parameters involved in the effective 2D Hamiltonian for the three-layer InAs/GaInSb and double HgTe/CdHgTe QWs considered in the main text.

SUPPLEMENTARY TEXT

A. An effective four-band model for cubic-semiconductor quantum well grown along $[\mathbf{0}mn]$ direction

A correct theoretical description of 2D systems with double band inversion is possible only on the basis of a Hamiltonian, which takes into account at least four bands. The minimal required effective 2D four-band Hamiltonian was first proposed for (001) double HgTe/CdHgTe QWs [1]. It was derived from the eight-band Kane model for the envelope wave functions of the Γ_6, Γ_8 and Γ_7 bands by preserving the full rotational symmetry in the QW plane [2, 3] and omitting the warping terms. In this section, we go beyond the axial approximation of Refs [1–3] and include the warping terms origin from cubic symmetry of diamond and zinc-blende semiconductors. The latter is essential for higher-order topology of cubic semiconductor QWs with double band inversion. In order to demonstrate that occurrence of the corner states depends on the growth orientation of 2D system, we further consider the general class of $(0mn)$-oriented QWs [4, 5] (m and n are integer numbers), which includes (001)-, (011)-, and (013)-oriented structures.

For simplicity, we limit ourselves to the upper 6×6 block of the Kane model including only the Γ_6 and Γ_8 bands. Although the Γ_7 band is needed for quantitative description of the positions of electron-like subbands in narrow QWs (see Supplemental material of Ref. [4]), it can be ignored for qualitative description of the band structure. We note that taking into account the contribution of the Γ_7 band does not lead to any new terms and just complicates the derivation of the effective 2D Hamiltonian (cf. Supplemental materials of Ref. [1] and Ref. [6]). Further, we also neglect the terms breaking inversion symmetry of 2D systems. These terms result from (i) the absence of inversion center in the unit cell of zinc-blende semiconductors; (ii) from the anisotropy of chemical bonds at the QW interfaces leading to the interface inversion asymmetry; and (iii) from the structural inversion asymmetry (SIA) in the growth direction. The latter arises only if the QW profile is asymmetric.

In the basis set of Bloch amplitudes in the sequence $|\Gamma_6,+1/2\rangle$,$|\Gamma_6,-1/2\rangle$, $|\Gamma_8,+3/2\rangle$, $|\Gamma_8,+1/2\rangle$, $|\Gamma_8,-1/2\rangle$, $|\Gamma_8,-3/2\rangle$ [4], the 6-band $\mathbf{k}\cdot\mathbf{p}$ Hamiltonian preserving inversion asymmetry is given by

$$H_{3D} = \begin{pmatrix} H_{cc} & H_{cv} \\ H_{cv}^{\dagger} & H_{vv} \end{pmatrix}, \tag{1}$$

where the blocks H_{cc} and H_{vv} represent the contribution from the Γ_6 and Γ_8 bands, respectively, and the block H_{cv} and the block H_{cv} describes the band mixing. The block H_{cc} is given by

$$H_{cc} = \left[E_c(z) + \frac{\hbar^2 \mathbf{k}\left[2F(z)+1\right]\mathbf{k}}{2m_0} + \Xi_c \mathrm{Tr}\epsilon\right] I_{2\times2}, \tag{2}$$

where $I_{2\times2}$ is the 2×2 identity matrix, $E_c(z)$ is the conduction band profile, $\mathbf{k} = (k_x, k_y, k_z)$ (note that $k_z = -i\partial/\partial z$ as z is the growth direction), $F(z)$ is a parameter accounting for contribution from remote bands, Ξ_c is the Γ_6-band deformation potential constant, and ϵ is the train tensor arising due to lattice-mismatch in the QW layers and the sample substrate. The block H_{cv} has the form

$$H_{cv} = \begin{pmatrix} -\dfrac{\sqrt{2}Pk_+}{2} & \dfrac{\sqrt{6}Pk_z}{3} & \dfrac{\sqrt{6}Pk_-}{6} & 0 \\ 0 & -\dfrac{\sqrt{6}Pk_+}{6} & \dfrac{\sqrt{6}Pk_z}{3} & \dfrac{\sqrt{2}Pk_-}{2} \end{pmatrix}, \tag{3}$$

where P is the Kane matrix element, $k_\pm = k_x \pm i k_y$. The block H_{vv} is given by

$$H_{vv} = E_v(z) I_{4\times4} + H_L^{(i)} + H_L^{(a)} + H_{BP}^{(i)} + H_{BP}^{(a)}, \tag{4}$$

where $I_{4\times4}$ is the 4×4 identity matrix, $E_v(z)$ is the valence band profile, $H_L^{(i)}$, $H_L^{(a)}$, $H_{BP}^{(i)}$ and $H_{BP}^{(a)}$ are the isotropic and anisotropic parts of the Luttinger and Bir-Pikus Hamiltonians,

$$H_L^{(i)} = \frac{\hbar^2}{2m_0}\left[-\mathbf{k}\left(\gamma_1 + \frac{5}{2}\gamma_2\right)\mathbf{k} + 2(\mathbf{J}\cdot\mathbf{k})\gamma_2(\mathbf{J}\cdot\mathbf{k})\right],$$
$$H_{BP}^{(i)} = \left(a + \frac{5}{4}b\right)\mathrm{Tr}\epsilon - b\sum_{\alpha} J_\alpha^2 \epsilon_{\alpha\alpha} - b\sum_{\alpha\neq\beta}\{J_\alpha, J_\beta\}_s \epsilon_{\alpha\beta}, \tag{5}$$

γ_1, γ_2, and γ_3 are contributions to the Luttinger parameters from remote bands; $\mathbf{J}$ is the vector composed of the matrices of the angular momentum 3/2; $\{J_\alpha, J_\beta\}_s = (J_\alpha J_\beta + J_\beta J_\alpha)/2$; a, b, and d are the Γ_8-band deformation potential constants.

In the coordinate frame relevant to $(0mn)$-oriented QWs $x \parallel [100]$, $y \parallel [0n\bar{m}]$, and $z \parallel [0mn]$ the terms $H_L^{(a)}$ and $H_{BP}^{(a)}$ assume the form [4, 5]:

$$H_L^{(a)} = \frac{\hbar^2}{2m_0}\Bigg(\{J_x, J_y\}_s(\gamma_3 - \gamma_2)k_x k_y + \{J_x, J_z\}_s\{\gamma_3 - \gamma_2, k_z\}_s k_x + \left[\{J_y, J_z\}_s \cos 2\theta - \frac{J_z^2 - J_y^2}{2}\sin 2\theta\right]\left[\{\gamma_3 - \gamma_2, k_z\}_s k_y \cos 2\theta - \frac{k_z(\gamma_3 - \gamma_2)k_z - (\gamma_3 - \gamma_2)k_y^2}{2}\sin 2\theta\right]\Bigg),$$

$$H_{BP}^{(a)} = -2\left(\frac{d}{\sqrt{3}} - b\right)\Bigg(\{J_x, J_y\}_s\epsilon_{xy} + \{J_x, J_z\}_s\epsilon_{xz} + \left[\{J_y, J_z\}_s \cos 2\theta - \frac{J_z^2 - J_y^2}{2}\sin 2\theta\right]\left[\epsilon_{yz}\cos 2\theta - \frac{\epsilon_{zz} - \epsilon_{yy}}{2}\sin 2\theta\right]\Bigg), \quad (6)$$

where $\theta = \arctan(m/n)$ is angle between the growth direction $[0mn]$ and the [001] axis. Expressions for the strain tensor components $\epsilon_{\alpha\beta}$ for the $(0mn)$-oriented QWs are provided in the Supplemental materials of Ref. [4].

We now derive an effective four-band 2D Hamiltonian for zinc-blende QW. Following the procedure described in Refs. [6, 7], we split H_{3D} in Eq. (1) into two parts

$$H_{3D} = H_0(k_z) + H_1(k_z, k_x, k_y, \theta), \quad (7)$$

where $H_0(k_z)$ preserves the inversion symmetry $\mathbf{P}$ at $k_x = k_y = 0$ and $\theta = 0$ and the full rotational symmetry in the QW plane, while $H_1(k_z, k_x, k_y, \theta)$ includes the rest terms.

First, we diagonalize the Hamiltonian $H_0(k_z)$ to obtain the energies and envelope functions of the QW states, as well as to classify of electronic levels as electron-like En, hole-like Hn or light-hole-like LHn levels ($n = 1, 2\ldots$). Since it is clear from the form of $H_0(k_z)$, the hole-like levels Hn at $k_x = k_y = 0$ are decoupled from the En and LHn. Therefore, the eigenfunctions of $H_0(k_z)$ are expanded in the basis of Bloch amplitudes as

$$|En,+\rangle = \begin{pmatrix} f_1^{(En)}(z)|\Gamma_6,+1/2\rangle \\ 0 \\ 0 \\ f_4^{(En)}(z)|\Gamma_8,+1/2\rangle \\ 0 \\ 0 \end{pmatrix}, \quad |En,-\rangle = \begin{pmatrix} 0 \\ f_2^{(En)}(z)|\Gamma_6,-1/2\rangle \\ 0 \\ 0 \\ f_5^{(En)}(z)|\Gamma_8,-1/2\rangle \\ 0 \end{pmatrix}, \quad |LHn,+\rangle = \begin{pmatrix} f_1^{(LHn)}(z)|\Gamma_6,+1/2\rangle \\ 0 \\ 0 \\ f_4^{(LHn)}(z)|\Gamma_8,+1/2\rangle \\ 0 \\ 0 \end{pmatrix},$$

$$|LHn,-\rangle = \begin{pmatrix} 0 \\ f_2^{(LHn)}(z)|\Gamma_6,-1/2\rangle \\ 0 \\ 0 \\ f_5^{(LHn)}(z)|\Gamma_8,-1/2\rangle \\ 0 \end{pmatrix}, \quad |Hn,+\rangle = \begin{pmatrix} 0 \\ 0 \\ f_3^{(Hn)}(z)|\Gamma_8,+3/2\rangle \\ 0 \\ 0 \\ 0 \end{pmatrix}, \quad |Hn,-\rangle = \begin{pmatrix} 0 \\ 0 \\ 0 \\ f_4^{(LHn)}(z)|\Gamma_8,-3/2\rangle \\ 0 \\ 0 \end{pmatrix}. \quad (8)$$

The presence of time reversal symmetry $\mathcal{T}$ and inversion symmetry $\mathbf{P}$ in $H_0(k_z)$ yields the relations [7]:

$$f_2^{(E\{2k+1\})} = \left(f_1^{(E\{2k+1\})}\right)^*, \qquad f_5^{(E\{2k+1\})} = -\left(f_4^{(E\{2k+1\})}\right)^*,$$
$$f_2^{(E\{2k+2\})} = -\left(f_1^{(E\{2k+2\})}\right)^*, \qquad f_5^{(E\{2k+2\})} = \left(f_4^{(E\{2k+2\})}\right)^*,$$
$$f_6^{(H\{2k+1\})} = \left(f_3^{(H\{2k+1\})}\right)^*,$$
$$f_6^{(H\{2k+2\})} = -\left(f_3^{(H\{2k+2\})}\right)^*,$$
$$f_2^{(LH\{2k+1\})} = -\left(f_1^{(LH\{2k+1\})}\right)^*, \qquad f_5^{(LH\{2k+1\})} = \left(f_4^{(LH\{2k+1\})}\right)^*,$$
$$f_2^{(LH\{2k+2\})} = \left(f_1^{(LH\{2k+2\})}\right)^*, \qquad f_5^{(LH\{2k+2\})} = -\left(f_4^{(LH\{2k+2\})}\right)^*.$$

Time reversal symmetry relates states with opposite spin; hence when the effective Hamiltonian for one spin is constructed, the Hamiltonian for the opposite spin can be easily obtained through the operation $\mathcal{T}$. The inversion operation $\mathbf{P}$ defines the parity of each subband. Since $H_0(k_z)$ preserves inversion symmetry, their eigenstates are also the eigenstates of the inversion operation $\mathbf{P}$, which can greatly simplify the calculation of the matrix elements of the effective 2D Hamiltonian. The parity of each subband is determined by both the envelope functions and the Bloch amplitudes at the Γ point. The parities of the envelope functions obtained by diagonalization of $H_0(k_z)$ [6, 7] are summarized in Table S1. The parities of the Bloch amplitudes are given by $\mathbf{P}|\Gamma_6, \pm 1/2\rangle = -|\Gamma_6, \pm 1/2\rangle$, $\mathbf{P}|\Gamma_8, \pm 1/2\rangle = |\Gamma_8, \pm 1/2\rangle$ and $\mathbf{P}|\Gamma_8, \pm 3/2\rangle = |\Gamma_8, \pm 3/2\rangle$. Thus, the parities of the QW subbands are $\mathbf{P}|E\{2k+1\}, \pm\rangle = -|E\{2k+1\}, \pm\rangle$, $\mathbf{P}|E\{2k+2\}, \pm\rangle = |E\{2k+2\}, \pm\rangle$, $\mathbf{P}|H\{2k+2\}, \pm\rangle = |H\{2k+2\}, \pm\rangle$, $\mathbf{P}|H\{2k+2\}, \pm\rangle = -|H\{2k+2\}, \pm\rangle$, $\mathbf{P}|LH\{2k+2\}, \pm\rangle = |LH\{2k+2\}, \pm\rangle$ and $\mathbf{P}|LH\{2k+2\}, \pm\rangle = -|LH\{2k+2\}, \pm\rangle$.

Next, we group the eigenstates of Eq. (8) into two classes. The first class, marked as class A, includes the basis states of our final effective 2D Hamiltonian $|E1\pm\rangle$, $|H1\pm\rangle$, $|H2\pm\rangle$ and $|E2\pm\rangle$. In the second class, denoted as class B, we consider all other subbands of the QW. The states in both classes are not coupled, since they are eigenstates of Hamiltonian $H_0(k_z)$. However, the presence of $H_1(k_z, k_x, k_y, \theta)$ introduces the mixing between the states from classes A and B. To derive the effective 2D Hamiltonian, we treat $H_1(k_z, k_x, k_y, \theta)$ as a small perturbation and perform a unitary transformation [8] to eliminate the coupling between the states from different classes by applying the second-order perturbation formula

$$H_{2D}(k_x, k_y)_{m,m'} = E_m \delta_{m,m'} + H'_{m,m'} + \frac{1}{2} \sum_l H'_{m,l} H'_{l,m'} \left(\frac{1}{E_m - E_l} + \frac{1}{E_{m'} - E_l} \right), \tag{9}$$

where

$$E_m = \int_{-\infty}^{+\infty} dz \sum_{\alpha,\beta=1}^{6} f_\alpha^{(m)}(z)^* \left(H_0(k_z)\right)_{\alpha,\beta} f_\beta^{(m')}(z),$$

$$H'_{m,m'} = \int_{-\infty}^{+\infty} dz \sum_{\alpha,\beta=1}^{6} f_\alpha^{(m)}(z)^* \left(H_1(k_z, k_x, k_y, \theta)\right)_{\alpha,\beta} f_\beta^{(m')}(z). \tag{10}$$

Here, the summation indices m, m' correspond to the states in class A, while the index l is for the states in class B. The Greek indices label envelope function component of $H_0(k_z)$. We note that accounting for the parity of the envelope functions $f_\alpha^{(m)}(z)$ given in Table S1 greatly simplifies calculation of $H'_{m,m'}$ [7].

Ordering the basis states as $|E1+\rangle$, $|H1+\rangle$, $|H2-\rangle$, $|E2-\rangle$, $|E1-\rangle$, $|H1-\rangle$, $|H2+\rangle$, $|E2+\rangle$, after calculating the matrix-elements in Eqs. (9) and (10), we are left with the effective 2D Hamiltonian in the form:

$$H_{2D}(k_x, k_y, \theta) = \begin{pmatrix} H_{4\times4}(k_x, k_y, \theta) & 0 \\ 0 & H^*_{4\times4}(-k_x, -k_y, \theta) \end{pmatrix}. \tag{11}$$

The diagonal blocks $H_{4\times4}(k_x, k_y, \theta)$ and $H^*_{4\times4}(-k_x, -k_y, \theta)$, in their turn, $H_{4\times4}(\mathbf{k}, \theta)$ can be split into isotropic and anisotropic parts:

$$H_{4\times4}(k_x, k_y, \theta) = H^{(i)}_{4\times4}(k_x, k_y, \theta) + H^{(a)}_{4\times4}(k_x, k_y, \theta). \tag{12}$$

The isotropic part $H^{(i)}_{4\times4}(k_x, k_y, \theta)$ is written as [2, 3]:

$$H^{(i)}_{4\times4}(k_x, k_y, \theta) = \begin{pmatrix} \epsilon_{E1}(k_x, k_y) & -A_1 k_+ & R_1^{(i)} k_-^2 & S_0 k_- \\ -A_1 k_- & \epsilon_{H1}(k_x, k_y) & 0 & R_2^{(i)} k_-^2 \\ R_1^{(i)} k_+^2 & 0 & \epsilon_{H2}(k_x, k_y) & A_2 k_+ \\ S_0 k_+ & R_2^{(i)} k_+^2 & A_2 k_- & \epsilon_{E2}(k_x, k_y) \end{pmatrix}, \tag{13}$$

where

$$\begin{aligned} \epsilon_{E1}(k_x, k_y) &= C_1 + M_1 - (D_1 + B_1)(k_x^2 + k_y^2), \\ \epsilon_{H1}(k_x, k_y) &= C_1 - M_1 - (D_1 - B_1)(k_x^2 + k_y^2), \\ \epsilon_{E2}(k_x, k_y) &= C_2 + M_2 - (D_2 + B_2)(k_x^2 + k_y^2), \\ \epsilon_{H2}(k_x, k_y) &= C_2 - M_2 - (D_2 - B_2)(k_x^2 + k_y^2), \\ C_2 &= C_1 + \frac{\Delta_{E1E2} - \Delta_{H1H2}}{2}. \end{aligned} \tag{14}$$

In Eqs (13) and (14), $C_{1,2}$, $M_{1,2}$, $A_{1,2}$, $B_{1,2}$, $D_{1,2}$, S_0 and $R^{(i)}_{1,2}$ are the structure parameters, which depend on θ (the growth direction of the QW), the QW profile and external conditions (such as temperature or hydrostatic pressure); Δ_{E1E2} and Δ_{H1H2} are the gaps between the $E1$ and $E2$ subbands and the $H1$ and $H2$ subbands, respectively.

The anisotropic term $H^{(a)}_{4\times4}(k_x, k_y, \theta)$ due to cubic symmetry of $H^{(a)}_L$ (see Eq. (6)) has the form

$$H^{(a)}_{4\times4}(k_x,k_y,\theta) = \begin{pmatrix} 0 & 0 & -R^{(a)}_1 k_+^2 & 0 \\ 0 & 0 & 0 & -R^{(a)}_2 k_+^2 \\ -R^{(a)}_1 k_-^2 & 0 & 0 & 0 \\ 0 & -R^{(a)}_2 k_-^2 & 0 & 0 \end{pmatrix} + \\ + \sin^2 2\theta \begin{pmatrix} 0 & 0 & -R^{(a)}_1 k_y^2 & 0 \\ 0 & 0 & 0 & -R^{(a)}_2 k_y^2 \\ -R^{(a)}_1 k_y^2 & 0 & 0 & 0 \\ 0 & -R^{(a)}_2 k_y^2 & 0 & 0 \end{pmatrix} + \\ + \sin 2\theta \begin{pmatrix} 0 & 0 & \tilde{R}^{(a)}_1(\theta) & 0 \\ 0 & 0 & 0 & \tilde{R}^{(a)}_2(\theta) \\ \tilde{R}^{(a)}_1(\theta) & 0 & 0 & 0 \\ 0 & \tilde{R}^{(a)}_2(\theta) & 0 & 0 \end{pmatrix}. \quad (15)$$

In Eqs. (13) and (15), $R^{(i)}_{1,2}$, $R^{(a)}_{1,2}$ and $\tilde{R}^{(a)}_{1,2}(\theta)$ are defined as

$$\tilde{R}^{(a)}_{1,2}(\theta) = \int\limits_{-\infty}^{+\infty} f^{(E1,H1)}_{4,3} \left[\left(k_z \frac{\sqrt{3}\hbar^2 \left(\gamma_3(z) - \gamma_2(z)\right)}{m_0} k_z - \left(\sqrt{3}b - d\right) (\epsilon_{xx} - \epsilon_{zz}) \right) \frac{\sin 2\theta}{4} + \left(\sqrt{3}b - d\right) \epsilon_{yz} \frac{\cos 2\theta}{2} \right] f^{(H2,E2)}_{6,5} dz,$$

$$R^{(i)}_{1,2} = \frac{\hbar^2}{2m_0} \frac{\sqrt{3}}{2} \int\limits_{-\infty}^{+\infty} f^{(E1,H1)}_{4,3} \left(\gamma_3(z) + \gamma_2(z)\right) f^{(H2,E2)}_{6,5} dz,$$

$$R^{(a)}_{1,2} = \frac{\hbar^2}{2m_0} \frac{\sqrt{3}}{2} \int\limits_{-\infty}^{+\infty} f^{(E1,H1)}_{4,3} \left(\gamma_3(z) - \gamma_2(z)\right) f^{(H2,E2)}_{6,5} dz. \quad (16)$$

Note that $\gamma_2(z)$ and $\gamma_3(z)$ are the symmetric functions of z in accordance with the assumption made above. One should recall that the strain tensor components $\epsilon_{\alpha\beta}$ also depend on θ; their expressions for the $(0mn)$-oriented QWs can be found in the Supplemental materials of Ref. [4].

Up to now, it has been assumed that x and y axis are oriented along (100) and $(0n\bar{m})$ crystallographic directions, respectively. To this end, we consider the edge in an arbitrary direction x', which has the angle φ relative to the x axis. To write the Hamiltonian in another coordinate system, where the z' and z axis coincide with $[0mn]$ crystallographic orientation, one should rotate the electron momentum according to the transformation:

$$\begin{pmatrix} k_x \\ k_y \end{pmatrix} = \begin{pmatrix} \cos\varphi & -\sin\varphi \\ \sin\varphi & \cos\varphi \end{pmatrix} \begin{pmatrix} k'_x \\ k'_y \end{pmatrix}. \quad (17)$$

Simultaneously with the transition from k'_x and k'_y to k_x and k_y, one should also apply a unitary transformation to the Hamiltonian (12):

$$H'_{4\times4}(k'_x, k'_y, \theta, \varphi) = U(\varphi) H_{4\times4}(k_x, k_y, \theta) U(\varphi)^{-1}, \quad (18)$$

where

$$U(\varphi) = \begin{pmatrix} 1 & 0 & 0 & 0 \\ 0 & e^{i\varphi} & 0 & 0 \\ 0 & 0 & e^{-2i\varphi} & 0 \\ 0 & 0 & 0 & e^{-i\varphi} \end{pmatrix}. \quad (19)$$

As it has been expected, $H'^{(i)}_{4\times4}(k'_x, k'_y, \theta)$ has the same form as $H^{(i)}_{4\times4}(k_x, k_y, \theta)$ in Eq. (13), while $H'^{(a)}_{4\times4}(k'_x, k'_y, \theta, \varphi)$ becomes

$$H'^{(a)}_{4\times4}(k'_x, k'_y, \theta, \varphi) = \begin{pmatrix} 0 & 0 & -R_1^{(a)} e^{i4\varphi} k'^2_+ & 0 \\ 0 & 0 & 0 & -R_2^{(a)} e^{i4\varphi} k'^2_+ \\ -R_1^{(a)} e^{-i4\varphi} k'^2_- & 0 & 0 & 0 \\ 0 & -R_2^{(a)} e^{-i4\varphi} k'^2_- & 0 & 0 \end{pmatrix} +$$
$$+ \left(k'_y \cos\varphi + k'_x \sin\varphi\right)^2 \sin^2 2\theta \begin{pmatrix} 0 & 0 & -R_1^{(a)} e^{i2\varphi} & 0 \\ 0 & 0 & 0 & -R_2^{(a)} e^{i2\varphi} \\ -R_1^{(a)} e^{-i2\varphi} & 0 & 0 & 0 \\ 0 & -R_2^{(a)} e^{-i2\varphi} & 0 & 0 \end{pmatrix} +$$
$$+ \sin 2\theta \begin{pmatrix} 0 & 0 & \tilde{R}_1^{(a)}(\theta) e^{i2\varphi} & 0 \\ 0 & 0 & 0 & \tilde{R}_2^{(a)}(\theta) e^{i2\varphi} \\ \tilde{R}_1^{(a)}(\theta) e^{-i2\varphi} & 0 & 0 & 0 \\ 0 & \tilde{R}_2^{(a)}(\theta) e^{-i2\varphi} & 0 & 0 \end{pmatrix}. \quad (20)$$

As expected from symmetry considerations, $H'^{(a)}_{4\times4}(k'_x, k'_y, \theta, \varphi)$ in Eq. (20) for (001)- and (010)-oriented QWs features a $\pi/2$-periodicity, i.e., $H'^{(a)}_{4\times4}(k'_x, k'_y, 0, \varphi \pm \pi/2) = H'^{(a)}_{4\times4}(k'_x, k'_y, 0, \varphi)$ and $H'^{(a)}_{4\times4}(k'_x, k'_y, \pi/2, \varphi \pm \pi/2) = H'^{(a)}_{4\times4}(k'_x, k'_y, \pi/2, \varphi)$. This is clear from Eqs (16), which show that $R^{(0)}_{1,2}(0) = R^{(0)}_{1,2}(\pm\pi/2) = 0$.

Further, we omit the prime marks keeping in mind that orientation of new x and y axis does not coincide with the crystallographic directions in the most general case. Parameters involved in $H^{(a)}_{4\times4}(k_x, k_y, \theta)$ and $H^{(a)}_{4\times4}(k_x, k_y, \theta, \varphi)$ for the semiconductor QWs considered in the main text are provided in Table S2.

B. Low-energy 1D Hamiltonian for the edge states in the $(0mn)$ QWs

To analyze the corner states in the QWs with double band inversion, we analytically derive the effective 1D Hamiltonian for the edge states. First, we split $H_{2D}(k_x, k_y, \theta, \varphi)$ (also see Eq. (11)) into two terms so that the first term represent two independent BHZ-like models for the pairs of $E1$-$H1$ subbands and $E2$-$H2$ subbands, while the second term includes the inter-pairs mixing. Then, assuming the open-boundary conditions in a semi-infinite plane $y > 0$, we solve the eigenvalue problems for independent BHZ-like blocks with $M_1 < 0$ and $M_2 < 0$ to find four wave functions at zero-wave vector along the boundary. Finally, we construct low-energy 1D Hamiltonian by projecting $H_{2D}(k_x, k_y, \theta, \varphi)$ onto the obtained set of the basis functions.

The edge wave functions (at zero wave-vector along the boundary) for two independent BHZ-like blocks for $E1$-$H1$ and $E2$-$H2$ subbands in $H_{2D}(k_x, k_y, \theta, \varphi)$ are written as:

$$|1,+\rangle = \frac{g_1(y)}{\sqrt{1+\eta_1^2}} \begin{pmatrix} 1 \\ \eta_1 \\ 0 \\ 0 \\ 0 \\ 0 \\ 0 \\ 0 \end{pmatrix}, \quad |2,-\rangle = \frac{g_2(y)}{\sqrt{1+\eta_2^2}} \begin{pmatrix} 0 \\ 0 \\ \eta_2 \\ 1 \\ 0 \\ 0 \\ 0 \\ 0 \end{pmatrix}, \quad |1,-\rangle = \frac{g_1(y)}{\sqrt{1+\eta_1^2}} \begin{pmatrix} 0 \\ 0 \\ 0 \\ 0 \\ 1 \\ \eta_1 \\ 0 \\ 0 \end{pmatrix}, \quad |2,+\rangle = \frac{g_2(y)}{\sqrt{1+\eta_2^2}} \begin{pmatrix} 0 \\ 0 \\ 0 \\ 0 \\ 0 \\ 0 \\ \eta_2 \\ 1 \end{pmatrix}, \quad (21)$$

where

$$\eta_n^2 = \frac{B_n + D_n}{B_n - D_n},$$
$$g_n(y) = N_n\left(\lambda_n^{(I)}, \lambda_n^{(II)}\right)\left\{e^{-\lambda_n^{(I)} y} - e^{-\lambda_n^{(II)} y}\right\},$$
$$N_n\left(\lambda_n^{(I)}, \lambda_n^{(II)}\right) = \sqrt{\left|2\lambda_n^{(I)}\lambda_n^{(II)} \frac{\lambda_n^{(I)} + \lambda_n^{(II)}}{\left(\lambda_n^{(I)} - \lambda_n^{(II)}\right)^2}\right|}. \quad (22)$$

Here, $n = 1$ and 2 correspond to the pairs of $E1$-$H1$ and $E2$-$H2$ subbands, respectively. In Eqs (22), while $\lambda_n^{(I)}$ and $\lambda_n^{(II)}$ have the form

$$\lambda_n^{(I,II)} = \sqrt{F_n \pm \sqrt{F_n^2 - \frac{M_n^2}{B_n^2}}},$$
$$F_n = \frac{A_n^2}{2(B_n^2 - D_n^2)} - \frac{M_n}{B_n}. \quad (23)$$

It is clear that for the existence of the edge states, $\lambda_n^{(I)}$ and $\lambda_n^{(II)}$ should have a non-zero real part. This can be achieved if $\lambda_n^{(I,II)}$ are both real (at $F_n^2 \geq M_n^2/B_n^2$). In the opposite case when $M_n^2/B_n^2 > F_n^2$, the square of $\lambda_n^{(I,II)}$ are complex conjugated:

$$\left\{\lambda_n^{(I,II)}\right\}^2 = F_n \pm i\sqrt{\frac{M_n^2}{B_n^2} - F_n^2}.$$

To go further, it is convenient to present the square root from complex number $Q + iW$ in algebraic form $\sqrt{Q + iW} = \pm(a + ib)$, where

$$a = \sqrt{\frac{\sqrt{Q^2 + W^2} + Q}{2}},$$
$$b = \mathrm{sgn}(W)\sqrt{\frac{\sqrt{Q^2 + W^2} - Q}{2}}.$$

This allows presenting $\lambda_n^{(I)}$ and $\lambda_n^{(II)}$ in the form $\lambda_n^{(I,II)} = a_n \pm ib_n$, where

$$a_n = \frac{\sqrt{2}}{2}\sqrt{\frac{M_n}{B_n} + F_n},$$
$$b_n = \frac{\sqrt{2}}{2}\sqrt{\frac{M_n}{B_n} - F_n}.$$

For the latter case, $g_n(y)$ in Eq. (22) can be written as

$$g_n(y) = 2\frac{\sqrt{|a_n|(a_n^2 + b_n^2)}}{|b_n|} e^{-a_n y} \sin(b_n y), \quad (24)$$

where a_n and b_n are both real being defined as $\lambda_n^{(I,II)} = a_n \pm b_n$.

Since we have set $k_y = -i\partial/\partial y$ to obtain Eqs. (21) and (22), we also need to introduce the following matrix elements:

$$\left\langle k_y^2 \right\rangle_{nm} = \int_0^{+\infty} g_n(y)\left(-\frac{\partial^2}{\partial y^2}\right) g_m(y) dy,$$
$$\left\langle k_y \right\rangle_{nm} = \int_0^{+\infty} g_n(y)\left(-i\frac{\partial}{\partial y}\right) g_m(y) dy. \quad (25)$$

A straightforward calculation results in

$$\left\langle k_y^2 \right\rangle_{nm} = \left\langle k_y^2 \right\rangle_{mn} = F(n,m)\left(\lambda_n^{(I)}\lambda_n^{(II)}\lambda_m^{(I)} + \lambda_n^{(I)}\lambda_m^{(I)}\lambda_m^{(II)} + \lambda_n^{(I)}\lambda_n^{(II)}\lambda_m^{(II)} + \lambda_n^{(II)}\lambda_m^{(I)}\lambda_m^{(II)}\right),$$
$$\left\langle k_y \right\rangle_{nm} = -\left\langle k_y \right\rangle_{mn} = -iF(n,m)\left(\lambda_n^{(I)}\lambda_n^{(II)} - \lambda_m^{(I)}\lambda_m^{(II)}\right), \quad (26)$$

where

$$F(n,m) = F(m,n) = N_n N_m \frac{\left(\lambda_n^{(I)} - \lambda_n^{(II)}\right)\left(\lambda_m^{(I)} - \lambda_m^{(II)}\right)}{\left(\lambda_n^{(I)} + \lambda_m^{(I)}\right)\left(\lambda_n^{(I)} + \lambda_m^{(II)}\right)\left(\lambda_n^{(II)} + \lambda_m^{(II)}\right)\left(\lambda_n^{(II)} + \lambda_m^{(I)}\right)} \quad (27)$$

One can see that $\langle k_y \rangle_{nn} = 0$ and $\langle k_y^2 \rangle_{nn} = \lambda_n^{(I)} \lambda_n^{(II)}$.

Before going further, we note that Eqs (26) and (27) are also valid for complex conjugated $\lambda_n^{(I,II)}$, i.e. for $\lambda_n^{(I,II)} = a_n \pm b_n$. For the latter case, Eqs (26) can be rewritten as

$$\langle k_y^2 \rangle_{nm} = \langle k_y^2 \rangle_{mn} = 8 \frac{\sqrt{|a_n|(a_n^2+b_n^2)}\sqrt{|a_m|(a_m^2+b_m^2)}\left(a_n^2 a_m + a_n a_m^2 + a_n b_m^2 + a_m b_n^2\right)}{\left[(a_n+a_m)^2+(b_n-b_m)^2\right]\left[(a_n+a_m)^2+(b_n+b_m)^2\right]},$$

$$\langle k_y \rangle_{nm} = -\langle k_y \rangle_{mn} = -4i \frac{\sqrt{|a_n|(a_n^2+b_n^2)}\sqrt{|a_m|(a_m^2+b_m^2)}\left(a_n^2 - a_m^2 + b_n^2 - b_m^2\right)}{\left[(a_n+a_m)^2+(b_n-b_m)^2\right]\left[(a_n+a_m)^2+(b_n+b_m)^2\right]}.$$

Since projection of the blocks $H_{4\times4}(k_x, k_y, \theta, \varphi)$ and $H^*_{4\times4}(-k_x, -k_y, \theta, \varphi)$ onto the edge basis functions in Eq. (21) is performed independently, we further focus on the upper block $H_{4\times4}(k_x, k_y, \theta, \varphi)$ only. The procedure for the lower block $H^*_{4\times4}(-k_x, -k_y, \theta, \varphi)$ can be done in a similar manner. Thus, to project $H_{4\times4}(k_x, k_y)$, one has to consider only the states $|1, +\rangle$ and $|2, -\rangle$, which are reduced to

$$|1\rangle = \frac{g_1(y)}{\sqrt{1+\eta_1^2}} \begin{pmatrix} 1 \\ \eta_1 \\ 0 \\ 0 \end{pmatrix}, \qquad |2\rangle = \frac{g_2(y)}{\sqrt{1+\eta_2^2}} \begin{pmatrix} 0 \\ 0 \\ \eta_2 \\ 1 \end{pmatrix}, \tag{28}$$

where the signs $\pm$ are omitted.

As mentioned above, for the projection of $H_{4\times4}(k_x, k_y, \theta, \varphi)$ onto the basis states $|1\rangle$ and $|2\rangle$, it is also convenient to present $H_{4\times4}(k_x, k_y, \theta, \varphi)$ in the form (cf. Eqs (13) and (20)):

$$H_{4\times4}(k_x, k_y, \theta, \varphi) = H^{(i)}_{2\times\mathrm{BHZ}}(k_x, k_y, \theta) + \widetilde{H}^{(a)}_{4\times4}(k_x, k_y, \theta, \varphi), \tag{29}$$

where

$$H^{(i)}_{2\times\mathrm{BHZ}}(k_x, k_y, \theta) = \begin{pmatrix} \epsilon_{E1}(k_x,k_y) & -A_1 k_+ & 0 & 0 \\ -A_1 k_- & \epsilon_{H1}(k_x,k_y) & 0 & 0 \\ 0 & 0 & \epsilon_{H2}(k_x,k_y) & A_2 k_+ \\ 0 & 0 & A_2 k_- & \epsilon_{E2}(k_x,k_y) \end{pmatrix}, \tag{30}$$

and

$$\begin{aligned} \widetilde{H}^{(a)}_{4\times4}(k_x, k_y, \theta, \varphi) = &\begin{pmatrix} 0 & 0 & R_1^{(i)} k_-^2 & S_0 k_- \\ 0 & 0 & 0 & R_2^{(i)} k_-^2 \\ R_1^{(i)} k_+^2 & 0 & 0 & 0 \\ S_0 k_+ & R_2^{(i)} k_+^2 & 0 & 0 \end{pmatrix} + \\ &+ \begin{pmatrix} 0 & 0 & -R_1^{(a)} e^{i4\varphi} k_+^2 & 0 \\ 0 & 0 & 0 & -R_2^{(a)} e^{i4\varphi} k_+^2 \\ -R_1^{(a)} e^{-i4\varphi} k_-^2 & 0 & 0 & 0 \\ 0 & -R_2^{(a)} e^{-i4\varphi} k_-^2 & 0 & 0 \end{pmatrix} + \\ &+ \left(k_y \cos\varphi + k_x \sin\varphi\right)^2 \sin^2 2\theta \begin{pmatrix} 0 & 0 & -R_1^{(a)} e^{i2\varphi} & 0 \\ 0 & 0 & 0 & -R_2^{(a)} e^{i2\varphi} \\ -R_1^{(a)} e^{-i2\varphi} & 0 & 0 & 0 \\ 0 & -R_2^{(a)} e^{-i2\varphi} & 0 & 0 \end{pmatrix} + \\ &+ \sin 2\theta \begin{pmatrix} 0 & 0 & \tilde{R}_1^{(a)}(\theta) e^{i2\varphi} & 0 \\ 0 & 0 & 0 & \tilde{R}_2^{(a)}(\theta) e^{i2\varphi} \\ \tilde{R}_1^{(a)}(\theta) e^{-i2\varphi} & 0 & 0 & 0 \\ 0 & \tilde{R}_2^{(a)}(\theta) e^{-i2\varphi} & 0 & 0 \end{pmatrix}. \end{aligned} \tag{31}$$

By using Eqs. (22)–(27) for integration along the y axis, the projection of $H^{(i)}_{2\times\mathrm{BHZ}}(k_x, k_y, \theta)$ leads to

$$H^{(i)}_{1D}(k_x, \theta) = \begin{pmatrix} C_1 - \dfrac{M_1 D_1}{B_1} - \dfrac{2A_1\eta_1}{1+\eta_1^2} k_x & 0 \\ 0 & C_2 - \dfrac{M_2 D_2}{B_2} + \dfrac{2A_2\eta_2}{1+\eta_2^2} k_x \end{pmatrix}. \tag{32}$$

Two blocks of $H_{1D}^{(i)}(k_x,\theta)$ corresponds to the edge states resulting from inversion of the subband pairs $|E1,+\rangle$–$|H1,+\rangle$ and $|E2,-\rangle$–$|H2,-\rangle$ in the absence of their mixing. Therefore, the energies of these edge states cross at $k_x = k_c$:

$$k_c = \frac{C_1 - C_2 + \dfrac{M_2 D_2}{B_2} - \dfrac{M_1 D_1}{B_1}}{\dfrac{2A_1\eta_1}{1+\eta_1^2} + \dfrac{2A_2\eta_2}{1+\eta_2^2}}. \tag{33}$$

Note that the crossing for other Kramer's partners occur at $k_x = -k_c$.

As clear from Eq. (32), the energy bands of $H_{1D}^{(i)}(k_x,\theta)$ are nothing but a tilted 1D Dirac cone. In this case, the projection of $\widetilde{H}_{4\times4}^{(a)}(k_x,k_y,\theta,\varphi)$ in Eq. (31) results in anti-diagonal mass terms describing the band-gap opening. After straightforward calculation, the matrix element $\langle 1|\widetilde{H}_{4\times4}^{(a)}(k_x,k_y,\theta,\varphi)|2\rangle$ is written as

$$\begin{aligned}\langle 1|\widetilde{H}_{4\times4}^{(a)}|2\rangle = \left(\left\langle k_y^2\right\rangle_{12} - k_x^2 - 2k_x\left\langle k_y\right\rangle_{12} i\right) e^{i4\varphi}F_a - \left(\left\langle k_y^2\right\rangle_{12} - k_x^2 + 2k_x\left\langle k_y\right\rangle_{12} i\right) F_i + \left(k_x - \left\langle k_y\right\rangle_{12} i\right) F_0 + \\ + e^{i2\varphi}\tilde{F}_a(\theta)\sin 2\theta - \left(\left\langle k_y^2\right\rangle_{12}\cos^2\varphi + k_x^2\sin^2\varphi + k_x\left\langle k_y\right\rangle_{12}\sin 2\varphi\right) e^{i2\varphi}F_a\sin^2 2\theta\end{aligned} \tag{34}$$

where

$$\begin{aligned}F_i &= \frac{R_1^{(i)}\eta_2 + R_2^{(i)}\eta_1}{\sqrt{1+\eta_1^2}\sqrt{1+\eta_2^2}},\\ F_a &= \frac{R_1^{(a)}\eta_2 + R_2^{(a)}\eta_1}{\sqrt{1+\eta_1^2}\sqrt{1+\eta_2^2}},\\ \tilde{F}_a(\theta) &= \frac{\tilde{R}_1^{(a)}(\theta)\eta_2 + \tilde{R}_2^{(a)}(\theta)\eta_1}{\sqrt{1+\eta_1^2}\sqrt{1+\eta_2^2}},\\ F_0 &= \frac{S_0}{\sqrt{1+\eta_1^2}\sqrt{1+\eta_2^2}}.\end{aligned} \tag{35}$$

The calculation of $\langle 2|\widetilde{H}_{4\times4}^{(a)}|1\rangle$ is performed in the same way.

On the basis of Eq. (26), the matrix elements of k_y can be presented in more convenient form

$$\begin{aligned}\left\langle k_y^2\right\rangle_{12} &= \left\langle k_y^2\right\rangle_{21} = \kappa_2,\\ \left\langle k_y\right\rangle_{12} &= -\left\langle k_y\right\rangle_{21} = -i\kappa_1.\end{aligned} \tag{36}$$

The latter allows writing projection of $\widetilde{H}_{4\times4}^{(a)}(k_x,k_y,\theta,\varphi)$ in the form

$$\begin{aligned}H_{1D}^{(a)}(k_x,\theta,\varphi) = \left[(F_i - F_a\cos 4\varphi)\,k_x^2 - (2F_i\kappa_1 + 2F_a\kappa_1\cos 4\varphi - F_0)\,k_x + F_a\kappa_2\cos 4\varphi - F_i\kappa_2 - F_0\kappa_1\right]\sigma_x + \\ + \left[\tilde{F}_a(\theta)\cos 2\varphi\sin 2\theta - F_a\sin^2 2\theta\left(k_x^2\cos 2\varphi\sin^2\varphi + \kappa_1 k_x\sin^2 2\varphi + \kappa_2\cos 2\varphi\cos^2\varphi\right)\right]\sigma_x + \\ + F_a\sin 4\varphi\left[k_x^2 + 2\kappa_1 k_x - \kappa_2\right]\sigma_y + \\ \left[-\tilde{F}_a(\theta)\sin 2\varphi\sin 2\theta + F_a\sin 2\varphi\sin^2 2\theta\left(k_x^2\sin^2\varphi - \kappa_1 k_x\cos 2\varphi + \kappa_2\cos^2\varphi\right)\right]\sigma_y.\end{aligned} \tag{37}$$

Expanding now $H_{1D}^{(i)}(k_x,\theta) + H_{1D}^{(a)}(k_x,\theta,\varphi)$ around $\delta k = k_x - k_c$, we finally obtain the low-energy effective Hamiltonian for the tilted gapped 1D fermions:

$$H_{\mathrm{1D}}(\delta k,\theta,\varphi) = \varepsilon_0 + v_0\delta k\mathbf{I}_2 + v_z\delta k\sigma_z + \left(m_y + v_y\delta k + \delta_y\delta k^2\right)\sigma_y + \left(m_x + v_x\delta k + \delta_x\delta k^2\right)\sigma_x, \tag{38}$$

where ε_0 is a constant corresponding to the energy of the crossing point at $k_x = k_c$ in the absence of $H_{1D}^{(a)}(k_x,\theta,\varphi)$,

$$\begin{aligned}v_0 &= \frac{A_1\eta_1}{1+\eta_1^2} - \frac{A_2\eta_2}{1+\eta_2^2},\\ v_z &= \frac{A_1\eta_1}{1+\eta_1^2} + \frac{A_2\eta_2}{1+\eta_2^2},\end{aligned}$$

$$m_x = (F_i - F_a\cos 4\varphi)\,k_c^2 + (F_0 - 2\kappa_1(F_i + F_a\cos 4\varphi))\,k_c + F_a\kappa_2\cos 4\varphi - F_i\kappa_2 - F_0\kappa_1 + \\ + \tilde{F}_a(\theta)\cos 2\varphi\sin 2\theta - F_a\sin^2 2\theta\left(k_c^2\cos 2\varphi\sin^2\varphi + \kappa_1 k_c\sin^2 2\varphi + \kappa_2\cos 2\varphi\cos^2\varphi\right),$$

$$\begin{aligned} m_y &= F_a\sin 4\varphi\left[k_c^2 + 2\kappa_1 k_c - \kappa_2\right] - \tilde{F}_a(\theta)\sin 2\varphi\sin 2\theta + F_a\sin 2\varphi\sin^2 2\theta\left(k_c^2\sin^2\varphi - \kappa_1 k_c\cos 2\varphi + \kappa_2\cos^2\varphi\right), \\ v_x &= F_0 + 2k_c\left(F_i - F_a\cos 4\varphi\right) - 2\kappa_1\left(F_i + F_a\cos 4\varphi\right) - F_a\sin^2 2\theta\left(2k_c\cos 2\varphi\sin^2\varphi + \kappa_1\sin^2 2\varphi\right), \\ v_y &= 2F_a\sin 4\varphi\left(\kappa_1 + k_c\right) + F_a\sin 2\varphi\sin^2 2\theta\left(2k_c\sin^2\varphi - \kappa_1\cos 2\varphi\right), \\ \delta_x &= F_i - F_a\cos 4\varphi - F_a\cos 2\varphi\sin^2\varphi\sin^2 2\theta, \\ \delta_y &= F_a\sin 4\varphi + F_a\sin 2\varphi\sin^2\varphi\sin^2 2\theta. \end{aligned} \quad (39)$$

The analogous calculations for the block $H^*_{4\times4}(-k_x, -k_y, \theta, \varphi)$ results in $H^*_{1\mathrm{D}}(-k_x - k_c, \theta, \varphi)$ (cf. Eq. (38)). The parameters of the effective 1D edge Hamiltonian $H_{1\mathrm{D}}(\delta k, \theta, \varphi)$ as a function of the edge orientation φ for the three-layer InAs/GaInSb and double HgTe/CdHgTe QWs considered in the main text are provided in Fig. S1.

C. Energy of 0D corner states

To calculate the energy of the corner states, we apply linear approximation for 1D edge 4×4 Hamiltonian consisting in two diagonal 2×2 blocks:

$$\begin{aligned} \tilde{H}^{(+)}_{1\mathrm{D}}(k, \theta, \varphi) &= \varepsilon_0 + v_0 k\mathbf{I}_2 + v_z k\sigma_z + (m_y + v_y k)\sigma_y + (m_x + v_x k)\sigma_x, \\ \tilde{H}^{(-)}_{1\mathrm{D}}(\tilde{k}, \theta, \varphi) &= \varepsilon_0 - v_0\tilde{k}\mathbf{I}_2 - v_z\tilde{k}\sigma_z - (m_y - v_y\tilde{k})\sigma_y + (m_x - v_x\tilde{k})\sigma_x, \end{aligned} \quad (40)$$

where $k = k_x - k_c$, $\tilde{k} = k_x + k_c$, $\tilde{H}^{(+)}_{1\mathrm{D}}(k, \theta, \varphi)$ and $\tilde{H}^{(-)}_{1\mathrm{D}}(\tilde{k}, \theta, \varphi)$ are nothing but linearized $H_{1\mathrm{D}}(k_x - k_c, \theta, \varphi)$ and $H^*_{1\mathrm{D}}(-k_x - k_c, \theta, \varphi)$, respectively (see Sec. B). Further, the constant ε_0 is omitted, while the eigenvalues of the 1D edge Hamiltonian are assumed to be counted from ε_0.

First, one should make a certain remark significantly simplifying the calculations. With the parameters given in Table S2, it is clear that both v_x and v_y are significantly lower than v_0 and v_z for any orientation of the edges (see Fig. S1). The straightforward calculations shows that the presence of v_x and v_y in Eq. (40) does not contribute significantly into the dispersion of the edge states as compared with other terms. Thus, one can neglect these terms in the first approximation and take them into account by using the perturbation theory.

Further, we focus on the upper block $\tilde{H}^{(+)}_{1\mathrm{D}}(k, \theta, \varphi)$, while the calculations for $\tilde{H}^{(-)}_{1\mathrm{D}}(\tilde{k}, \theta, \varphi)$ are performed in a similar way. Let us now make a unitary transformation of $\tilde{H}^{(+)}_{1\mathrm{D}}(k, \theta, \varphi)$ as follows $H^{(+)}_{1\mathrm{D}}(k, \theta, \varphi) = U_+\tilde{H}^{(+)}_{1\mathrm{D}}(k, \theta, \varphi)U_+^\dagger$, where

$$U_+ = \frac{1}{\sqrt{2}}\begin{pmatrix} 1 & i \\ -i & -1 \end{pmatrix}. \quad (41)$$

The straightforward calculations results in

$$H^{(+)}_{1\mathrm{D}}(k, \theta, \varphi) = v_0 k\mathbf{I}_2 - v_z k\sigma_y - m_y\sigma_z - m_x\sigma_x. \quad (42)$$

It is clear that $H^{(+)}_{1\mathrm{D}}(k, \theta, \varphi)$ represent a 1D Dirac Hamiltonian, modified by "tilted" term $v_0 k\mathbf{I}_2$ and additional mass term $m_x\sigma_x$.

Now, for a quantitative description, we define the coordinate x along the curved edge so that $x = 0$ corresponds to the meeting corner. In this case, m_x, m_y in Eq. (42) are the function of x, and $k \equiv \hat{k} = -i\partial/\partial x$. Under this assumption, $H^{(+)}_{1\mathrm{D}}(\hat{k}, \theta, \varphi)$ is defined in disjoint regions far from $x = 0$. To define the 1D system fully, one needs to specify the boundary conditions that the wave functions must satisfy in the vicinity of $x = 0$ in order to ensure that probability current along the curved edge is conserved. The current conservation implies that

$$\Phi_1^\dagger(v_0\mathbf{I}_2 - v_z\sigma_y)\Phi_1 = \Phi_2^\dagger(v_0\mathbf{I}_2 - v_z\sigma_y)\Phi_2, \quad (43)$$

where Φ_1 and Φ_2 are the wave-functions defined from different sides of the corner. Note that specific type of the corner has not yet been determined.

Let us now discuss the general *linear* boundary condition between Φ_1 and Φ_2. Let us assume that

$$\Phi_1\big|_{x=-\eta} = \Pi\Phi_2\big|_{x=+\eta}, \quad (44)$$

where η is a positive quantity and Π is a unitary 2×2 matrix. Then Eq. (43) will be satisfied if $\Pi^{\dagger}(v_0\mathbf{I}_2 - v_z\sigma_y)\Pi = v_0\mathbf{I}_2 - v_z\sigma_y$. The latter results in

$$\Pi = \exp\left\{-i(\beta\sigma_y + \gamma\mathbf{I}_2)\right\}, \tag{45}$$

where β and γ are real parameters. Note that changing $\beta \to \beta + \pi$ and $\gamma \to \gamma + \pi$ has no effect on any physical quantities since this is just equivalent to changing $\Phi_1 \to -\Phi_1$. Thus, one can assume that β and γ lie in the range from $-\pi/2$ to $\pi/2$.

The parameters β and γ in Eq. (45) can be given a precise physical interpretation. Let us consider an additional δ-function potential barrier in $H^{(+)}_{\rm 1D}(k,\theta,\varphi)$ placed at $x=0$ given by $V_0\delta(0)$, where V_0 is a real parameter. Then, by integrating the Schrödinger equation with the Hamiltonian in Eq. (42) through this potential, one can show that the wave function has indeed a discontinuity given by

$$\Phi_1\big|_{x=-\eta} = e^{-iV_0\left(\frac{v_z}{v_z^2-v_0^2}\sigma_y + \frac{v_0}{v_z^2-v_0^2}\mathbf{I}_2\right)}\Phi_2\big|_{x=+\eta}, \tag{46}$$

which coincides with Eqs (44) and (45) if one defines β and γ as

$$\beta = \frac{v_z}{v_z^2 - v_0^2}V_0, \qquad\qquad \gamma = \frac{v_0}{v_z^2 - v_0^2}V_0. \tag{47}$$

The discontinuity is not surprising. We recall that for conventional non-relativistic Schrödinger equation, which is second order in spatial derivatives, a δ-function potential barrier leads to a discontinuity in the first derivative of the wave function. For the Dirac-like Hamiltonian, which is first order in spatial derivative, a δ-function potential leads to a discontinuity in the wave function. Thus, Eqs (44) and (45) at non-zero β and γ include the effects of a thin (δ-like) barrier, which could possibly be present at the corner.

Representing β and γ as $\beta = \beta_2 - \beta_1$ and $\gamma = \gamma_2 - \gamma_1$, Eq. (44) can be written in the form

$$\Pi(\beta_1,\gamma_1)\Phi_1\big|_{x=-\eta} = \Pi(\beta_2,\gamma_2)\Phi_2\big|_{x=+\eta}. \tag{48}$$

Thus, by means of Eq. (45), one can write a new Hamiltonian $H^{(\rm new)}_{\rm 1D}(k,\tilde\beta,\tilde\gamma,\theta,\varphi) = \Pi(\tilde\beta,\tilde\gamma)H^{(+)}_{\rm 1D}(k,\theta,\varphi)\Pi^{\dagger}(\tilde\beta,\tilde\gamma)$ for the wave-functions $\Psi_{\rm 0D}(x) = \Pi(\tilde\beta,\tilde\gamma)\Phi$ that are continuous in the vicinity of $x=0$:

$$H^{(\rm new)}_{\rm 1D}(k,\tilde\beta,\theta,\tilde\gamma,\varphi) = v_0\hat{k}\mathbf{I}_2 - v_z\hat{k}\sigma_y + M_z(x)\sigma_z - M_x(x)\sigma_x, \tag{49}$$

where $M_z(x)$ and $M_x(x)$ are defined as

$$\begin{aligned} M_z(x) &= m_x(x)\sin 2\tilde\beta - m_y(x)\cos 2\tilde\beta, \\ M_x(x) &= m_x(x)\cos 2\tilde\beta + m_y(x)\sin 2\tilde\beta. \end{aligned} \tag{50}$$

One can see that $H^{(\rm new)}_{\rm 1D}(k,\tilde\beta,\tilde\gamma,\theta,\varphi)$ is actually independent of $\tilde\gamma$. Therefore, γ in Eqs. (45)–(48) can be set to zero, and the boundary conditions at the corner can be considered to be characterized only by $\tilde\beta$ dependent on x. This case is discussed in the main text.

In view of the above, the Schrödinger equation for the corner states takes the form

$$\left(-v_z\hat{k}\sigma_y + M_z(x)\sigma_z - M_x(x)\sigma_x\right)\Psi_{\rm 0D}(x) = \left(E - v_0\hat{k}\right)\mathbf{I}_2\Psi_{\rm 0D}(x). \tag{51}$$

Let us act by the matrix operator from the left-hand side of Eq. (51) on both sides of this equation. This leads to

$$\left\{\left(v_z^2\hat{k}^2 + M_z^2 + M_x^2 - (E - v_0\hat{k})^2\right)\mathbf{I}_2 + v_z\begin{pmatrix} -M_x' & -M_z' \\ -M_z' & M_x' \end{pmatrix} - v_0\begin{pmatrix} -iM_z' & iM_x' \\ iM_x' & iM_z' \end{pmatrix}\right\}\Psi_{\rm 0D}(x) = 0, \tag{52}$$

where the prime denotes the derivative with respect to x.

To find an exact solution of Eq. (52), we further restrict ourselves to the case, in which $M_z(x)$ and $M_x(x)$ are all proportional to each other

$$M_x(x) = \alpha M_z(x) + m, \tag{53}$$

where

$$\alpha = \frac{M_x(-\infty) - M_x(+\infty)}{M_z(-\infty) - M_z(+\infty)},$$
$$m = \frac{M_z(-\infty)M_x(+\infty) - M_z(+\infty)M_x(-\infty)}{M_z(-\infty) - M_z(+\infty)}. \tag{54}$$

We note that Eq. (53) is a good approximation only for the relatively sharp functions varying in the vicinity of $x = 0$. It is clear that Eq. (53) becomes *exact* in the limit of the step-like functions $M_x(x)$ and $M_z(x)$. The latter corresponds to the corner shown in Fig. 3 in the main text. Thus, Eq. (52) reads

$$\left\{ \left(v_z^2\hat{k}^2 + M_z(x)^2 + \{\alpha M_z(x) + m\}^2 - (E - v_0\hat{k})^2\right)\mathbf{I}_2 + M_z' \begin{pmatrix} -v_z\alpha + iv_0 & -v_z - iv_0\alpha \\ -v_z - iv_0\alpha & v_z\alpha - iv_0 \end{pmatrix} \right\}\Psi_{0\mathrm{D}}(x) = 0. \tag{55}$$

Hence solutions of Eq. (55) may be constructed as follows:

$$\Psi_{0\mathrm{D}}(x) = \chi\psi(x), \tag{56}$$

where χ is the spin part of the wave function satisfying equation

$$\begin{pmatrix} -v_z\alpha + iv_0 & -v_z - iv_0\alpha \\ -v_z - iv_0\alpha & v_z\alpha - iv_0 \end{pmatrix}\chi = \nu\chi,$$

with eigenvalues $\nu = \pm\sqrt{1+\alpha^2}\sqrt{v_z^2 - v_0^2}$.

The equation for the coordinate part $\psi(x)$ can be written as

$$\left\{ \left(\sqrt{v_z^2 - v_0^2}\hat{k} + \frac{Ev_0}{\sqrt{v_z^2 - v_0^2}}\right)^2 + \left(\sqrt{1+\alpha^2}M_z(x) + \frac{m\alpha}{\sqrt{1+\alpha^2}}\right)^2 - \frac{E^2v_z^2}{v_z^2 - v_0^2} + \frac{m^2}{1+\alpha^2} + \nu M_z' \right\}\psi(x) = 0. \tag{57}$$

Finally, by introducing a new variable $\tilde{x} = x/\sqrt{v_z^2 - v_0^2}$ and representing $\psi(x)$ in the form

$$\psi(x) = \tilde{\psi}(\tilde{x})e^{-i\tilde{x}\frac{Ev_0}{\sqrt{v_z^2 - v_0^2}}}, \tag{58}$$

we arrive at the following equation:

$$\left\{ \hat{\tilde{k}}^2 + \tilde{W}(\tilde{x})^2 + \sigma\tilde{W}(\tilde{x})' \right\}\tilde{\psi}(\tilde{x}) = \varepsilon\tilde{\psi}(\tilde{x}), \tag{59}$$

where $\sigma = \pm 1$ (the sign of σ coincides with those for ν), and ε and $\tilde{W}(\tilde{x})$ are defined as

$$\varepsilon = \frac{E^2v_z^2}{v_z^2 - v_0^2} - \frac{m^2}{1+\alpha^2},$$
$$\tilde{W}(\tilde{x}) = \sqrt{1+\alpha^2}M_z + \frac{m\alpha}{\sqrt{1+\alpha^2}}. \tag{60}$$

As seen from Eq. (59), it is the common Schrödinger equation with a specific potential, which is a linear combination of the square of the derivative of the same function $\tilde{W}(\tilde{x})$. It possesses a special symmetry and represents the formulation of supersymmetric quantum mechanics [9]. The supersymmetric potential $\tilde{W}(\tilde{x})$ allows for factorization of Eq. (59):

$$\left(-i\hat{\tilde{k}} - \sigma\tilde{W}(\tilde{x})\right)\left(i\hat{\tilde{k}} - \sigma\tilde{W}(\tilde{x})\right)\tilde{\psi}(\tilde{x}) = \varepsilon\tilde{\psi}(\tilde{x}), \tag{61}$$

If the signs of the asymptotics $\tilde{W}(+\infty)$ and $\tilde{W}(-\infty)$ are opposite, i.e.

$$\left(M_z(+\infty) + \frac{m\alpha}{1+\alpha^2}\right)\left(M_z(-\infty) + \frac{m\alpha}{1+\alpha^2}\right) < 0, \tag{62}$$

Eq. (61) always has a localized solution $\tilde{\psi}(\tilde{x})$ with $\varepsilon = 0$, which converts the second brackets into zero:

$$\left(\frac{d}{d\tilde{x}} - \sigma \tilde{W}(\tilde{x})\right) \tilde{\psi}(\tilde{x}) = 0. \tag{63}$$

Solution of this equation has the form

$$\tilde{\psi}(\tilde{x}) \sim e^{\sigma \int\limits_0^{\tilde{x}} \tilde{W}(z) dz}, \tag{64}$$

where the sign of σ should be chosen in accordance with normalized condition of $\tilde{\psi}(\tilde{x})$. If $\tilde{W}(+\infty) > 0$, $\sigma = -1$, while for $\tilde{W}(+\infty) < 0$, $\sigma = 1$. We must note that these two cases are not equivalent. One could see that the values of $\sigma = 1$ and $\sigma = -1$ correspond to the internal and external corners at the same positions of two edges. Further, we show that the localized states for these two corners have different energies.

By using Eqs. (56), (58), (60) and (64), the wave function of the corner state is expressed as follows:

$$\Psi_{0\mathrm{D}}(x) = C \begin{pmatrix} v_z \alpha - \sigma \sqrt{1+\alpha^2}\sqrt{v_z^2 - v_0^2} - i v_0 \\ v_z + i v_0 \alpha \end{pmatrix} e^{-ix \frac{E v_0}{v_z^2 - v_0^2} + \frac{\sigma}{\sqrt{1+\alpha^2}\sqrt{v_z^2 - v_0^2}} \int\limits_0^x \left\{(1+\alpha^2) M_z(z) + m\alpha\right\} dz}, \tag{65}$$

where C is the normalization constant. Now substituting $\Psi_{0\mathrm{D}}(x)$ into Eq. (51), one can show that

$$E = \frac{\sigma m}{\sqrt{1+\alpha^2}} \frac{\sqrt{v_z^2 - v_0^2}}{v_z}. \tag{66}$$

Thus, the localized states for the internal and external corners, corresponding to the same positions of two edges, have opposite energies. One can verified that

$$E^2 < \frac{m^2}{1+\alpha^2} \leq M_x(x)^2 + M_z(x)^2 = m_x(x)^2 + m_y(x)^2.$$

Let us make few remarks concerning the results obtained above. First, we have found a localized 0D corner state, whose energy does not depend on the specific type of functions $M_z(x)$ and $M_x(x)$. The existence of such localized state is guaranteed by the two conditions defined by Eq. (53) and Eq. (62). The latter can be also written in equivalent form

$$(M_z(+\infty) + \alpha M_x(+\infty))\,(M_z(-\infty) + \alpha M_x(-\infty)) < 0. \tag{67}$$

We now take into account the small terms previously neglected in Eq. (40). Note that before applying the perturbation theory on the basis of the wave-function given be Eq. (65), one should perform the unitary transformation to get the correction to $H_{1\mathrm{D}}^{(\mathrm{new})}(k, \tilde{\beta}, \theta, \varphi)$:

$$\delta H_{1\mathrm{D}}^{(\mathrm{new})}(k, \tilde{\beta}, \theta, \varphi) = \left(v_x(x)\sin 2\tilde{\beta} - v_y(x)\cos 2\tilde{\beta}\right) k\sigma_z - \left(v_x(x)\cos 2\tilde{\beta} + v_y(x)\sin 2\tilde{\beta}\right) k\sigma_x. \tag{68}$$

The first-order correction to the energy of the localized corner state in Eq. (66) can be calculated analytically assuming that $M_x(x)$ and $M_z(x)$ are the step-like functions, for which the theory above is *exact*. The straightforward calculations on the basis of $\Psi_{0\mathrm{D}}(x)$ leads to the first-order energy shift:

$$\delta E = -\frac{v_0 m}{v_z^2(1+\alpha^2)} \left(V(-\infty) \frac{\lambda(+\infty)}{\lambda(-\infty) + \lambda(\infty)} + V(+\infty) \frac{\lambda(-\infty)}{\lambda(-\infty) + \lambda(\infty)} \right), \tag{69}$$

where

$$V(x) = (v_x + \alpha v_y)\cos 2\tilde{\beta} + (v_y - \alpha v_x)\sin 2\tilde{\beta},$$
$$\lambda(\pm\infty) = \frac{(1+\alpha^2) M_z(\pm\infty) + m\alpha}{\sqrt{1+\alpha^2}\sqrt{v_z^2 - v_0^2}}. \tag{70}$$

* sergey.krishtopenko@gmail.com

[1] S. S. Krishtopenko, W. Knap, and F. Teppe, Sci. Rep. **6**, 30755 (2016).
[2] S. S. Krishtopenko and F. Teppe, Phys. Rev. B **97**, 165408 (2018).
[3] S. S. Krishtopenko, M. Antezza, and F. Teppe, Phys. Rev. B **101**, 205424 (2020).
[4] S. S. Krishtopenko, I. Yahniuk, D. B. But, V. I. Gavrilenko, W. Knap, and F. Teppe, Phys. Rev. B **94**, 245402 (2016).
[5] K.-M. Dantscher, D. A. Kozlov, P. Olbrich, C. Zoth, P. Faltermeier, M. Lindner, G. V. Budkin, S. A. Tarasenko, V. V. Bel'kov, Z. D. Kvon, N. N. Mikhailov, S. A. Dvoretsky, D. Weiss, B. Jenichen, and S. D. Ganichev, Phys. Rev. B **92**, 165314 (2015).
[6] B. A. Bernevig, T. L. Hughes, and S.-C. Zhang, Science **314**, 1757 (2006).
[7] D. G. Rothe, R. W. Reinthaler, C.-X. Liu, L. W. Molenkamp, S.-C. Zhang, and E. M. Hankiewicz, New J. Phys. **12**, 065012 (2010).
[8] R. Winkler, Spin-Orbit Coupling Effects in Two-Dimensional Electron and Hole Systems, Springer, Berlin, Heidelberg (2003).
[9] E. Witten, Nucl. Phys. B **188**, 513 (1981).

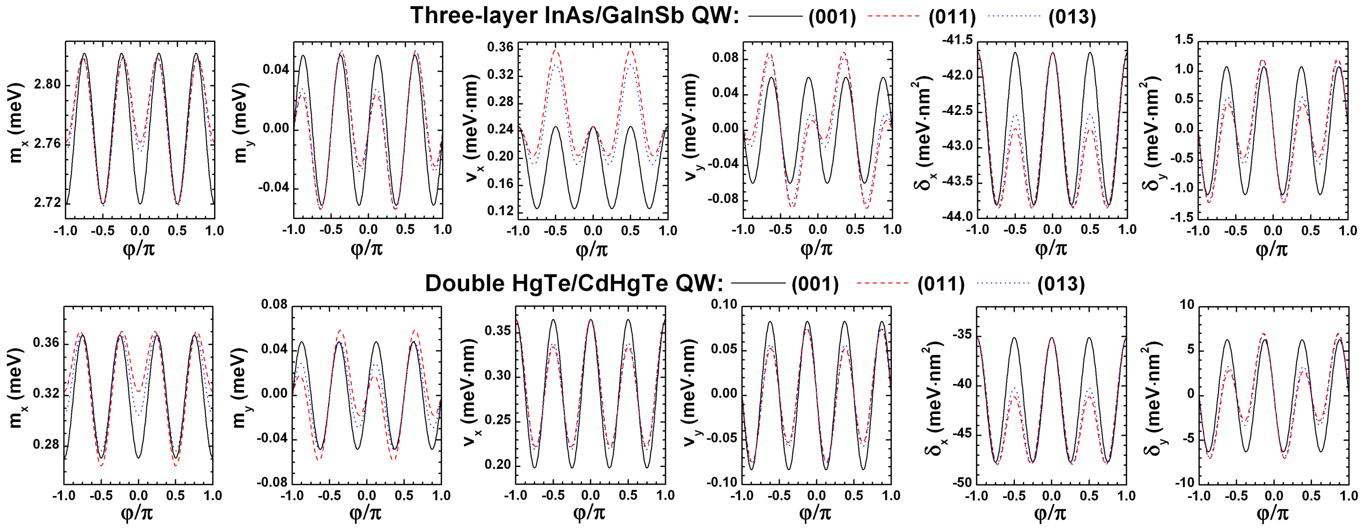

FIG. S1. Parameters of effective 1D edge Hamiltonian as a function of the edge orientation φ for the three-layer InAs/GaInSb and double HgTe/CdHgTe QWs considered in the main text at different growth orientations. The edge independent parameters (v_0, v_z) equal to (36.6, 68.1) meV·nm and (−16.9, 200.3) meV·nm for the three-layer InAs/GaInSb and double HgTe/CdHgTe QWs, respectively.

TABLE S1. Parities of the envelope functions of multi-band $\mathbf{k}\cdot\mathbf{p}$ Hamiltonian at zero electron momentum in the QW plane.

QW subband	$E\{2k+1\}$	$E\{2k+2\}$	$H\{2k+1\}$	$H\{2k+2\}$	$LH\{2k+1\}$	$LH\{2k+2\}$
Even	$f_{1,2}^{(E\{2k+1\})}(z)$	$f_{4,5}^{(E\{2k+2\})}(z)$	$f_{3,6}^{(H\{2k+1\})}(z)$	–	$f_{4,5}^{(LH\{2k+1\})}(z)$	$f_{1,2}^{(LH\{2k+2\})}(z)$
Odd	$f_{4,5}^{(E\{2k+1\})}(z)$	$f_{1,2}^{(E\{2k+2\})}(z)$	–	$f_{3,6}^{(H\{2k+2\})}(z)$	$f_{1,2}^{(LH\{2k+1\})}(z)$	$f_{4,5}^{(LH\{2k+2\})}(z)$

TABLE S2. Parameters involved in the effective 2D Hamiltonian for the three-layer InAs/GaInSb and double HgTe/CdHgTe QWs considered in the main text.

2D system	C_1 (meV)	C_2 (meV)	M_1 (meV)	M_2 (meV)	B_1 (meV·nm^2)	B_2 (meV·nm^2)	D_1 (meV·nm^2)	D_2 (meV·nm^2)	S_0 (meV·nm)
Three-layer InAs/GaInSb QW	51.60	34.93	-45.35	-20.78	-720	-520	-51	-320	-37
Double HgTe/CdHgTe QW	-37.90	-27.43	-13.99	-3.52	-1175	-695	-1025	-545	2

2D system	A_1 (meV·nm)	A_2 (meV·nm)	$R_1^{(i)}$ (meV·nm^2)	$R_2^{(i)}$ (meV·nm^2)	$R_1^{(a)}$ (meV·nm^2)	$R_2^{(a)}$ (meV·nm^2)
Three-layer InAs/GaInSb QW	105	40	-56	-27	-1.1	-1.3
Double HgTe/CdHgTe QW	375	350	-320	110	-12.0	-10.8

2D system	$\tilde{R}_1^{(a)}$ [001] (meV·nm)	$\tilde{R}_2^{(a)}$ [001] (meV·nm)	$\tilde{R}_1^{(a)}$ [011] (meV·nm)	$\tilde{R}_2^{(a)}$ [011] (meV·nm)	$\tilde{R}_1^{(a)}$ [013] (meV·nm)	$\tilde{R}_2^{(a)}$ [013] (meV·nm)
Three-layer InAs/GaInSb QW	0	0	$-3.6\cdot10^{-3}$	$-2.8\cdot10^{-4}$	$-1.6\cdot10^{-3}$	$-2.1\cdot10^{-4}$
Double HgTe/CdHgTe QW	0	0	$-2.4\cdot10^{-2}$	$-1.6\cdot10^{-3}$	$-1.4\cdot10^{-2}$	$-1.2\cdot10^{-3}$
